# *PIWI* genes and piRNAs are ubiquitously expressed in mollusks and show patterns of lineage-specific adaptation

**DOI:** 10.1038/s42003-018-0141-4

**Published:** 2018-09-07

**Authors:** Julia Jehn, Daniel Gebert, Frank Pipilescu, Sarah Stern, Julian Simon Thilo Kiefer, Charlotte Hewel, David Rosenkranz

**Affiliations:** Institute of Organismic and Molecular Evolution, Anthropology, Johannes Gutenberg University Mainz, Anselm-Franz-von-Bentzel-Weg 7, 55099 Mainz, Germany

## Abstract

PIWI proteins and PIWI-interacting RNAs (piRNAs) suppress transposon activity in animals, thus protecting their genomes from detrimental insertion mutagenesis. Here, we reveal that *PIWI* genes and piRNAs are ubiquitously expressed in mollusks, similar to the situation in arthropods. We describe lineage-specific adaptations of transposon composition in piRNA clusters in the great pond snail and the pacific oyster, likely reflecting differential transposon activity in gastropods and bivalves. We further show that different piRNA clusters with unique transposon composition are dynamically expressed during oyster development. Finally, bioinformatics analyses suggest that different populations of piRNAs presumably bound to different *PIWI* paralogs participate in homotypic and heterotypic ping-pong amplification loops in a tissue- and sex-specific manner. Together with recent findings from other animal species, our results support the idea that somatic piRNA expression represents the ancestral state in metazoans.

## Abstract (web summary)

Julia Jehn et al. report a phylogenetic analysis and expression studies of *PIWI* genes in 11 mollusk species. They find that *PIWI* genes and piRNAs are expressed in both the germline and soma, as in arthropods, suggesting that somatic expression of the piRNA system represents the ancestral state in animals.

## Introduction

In virtually all animals, PIWI proteins protect germ cells from the steady threat of mobile genetic elements, so-called transposons^1,2^. Based on sequence complementarity to their target transcripts, 23–31 nt non-coding RNAs, termed PIWI-interacting (pi-) RNAs, function as guide molecules for PIWI proteins that slice matching targets through their endonuclease activity. Besides post-transcriptional transposon control, PIWI proteins and piRNAs can trigger the establishment of repressive epigenetic DNA or chromatin modifications, thus inducing efficient transposon silencing on the transcriptional level^3–6^.

Analyses of piRNA pathways in representatives of many animal taxa have unveiled a great diversity of lineage-specific adaptations, challenging the universal validity of insights obtained from model organisms^7–19^. For a long time, PIWI proteins and piRNAs were thought to be dispensable for female germ cell development in mammals until it became clear that the model organisms mouse and rat represent an exception from the mammalian rule in that they employ an oocyte specific Dicer isoform for transposon control instead of Piwil3 which is expressed in the bovine and human female germline^15,20^. Similarly, evidence for a gene-regulatory role of piRNAs^14,21–27^ and their widespread somatic expression in many animals^19,28–35^ have eroded the dogma that the piRNA pathway is restricted to the germline, being exclusively responsible for silencing of transposons. Indeed, it has been shown that piRNAs are essential for regeneration and stem cell maintenance in the flatworm *Schmidtea mediterranea*^28^, provide an adaptive immunity against virus infections in *Aedes aegypti*^36^, are responsible for sex determination in *Bombyx mori*^37^ and memory-related synaptic plasticity in *Aplysia californica*^38^.

Despite the likely more than seventy thousand living molluskan species^39^ there exist only a few functional descriptions of PIWI proteins or piRNAs for this taxon based on experiments in the sea slug *Aplysia californica*^38^, the Farrer’s scallop *Chlamys farreri*^40^ and in the dog whelk *Nucella lapillus*^41^. Importantly, Waldron et al.^41^ recently showed that piRNA-like small RNAs matching virus and transposon sequences are somatically expressed in *Nucella lapillus*. However, the available data do not allow us to draw any conclusions on whether this represents a conserved or lineage-specific feature of the PIWI/piRNA system within mollusks. In order to further elucidate the evolution of the PIWI/piRNA system in mollusks, we have reconstructed the evolution of *PIWI* genes in this phylum based on 11 sequenced genomes showing that *Piwil1* and *Piwil2* are conserved in mollusks. We perform quantitative real-time PCR experiments to analyze the expression patterns of the identified *PIWI* paralogs across a representative set of tissues from the great pond snail *Lymnaea stagnalis* (*L. stagnalis*) and the pacific oyster *Crassostrea gigas* (*C. gigas*). We apply high-throughput sequencing of small RNAs from *L. stagnalis* to verify the presence of piRNAs in germline and muscle tissue. We further reanalyze published small RNA sequence data from *C. gigas* to characterize the dynamic expression of piRNAs from distinct piRNA clusters during oyster development. Finally, we use bioinformatics approaches to show that different piRNA populations and *PIWI* paralogs participate in the ping-pong amplification loop in a tissue-specific and sex-specific manner.

## Results

### The molluskan *PIWI* gene repertoire

**Fig. 1:**
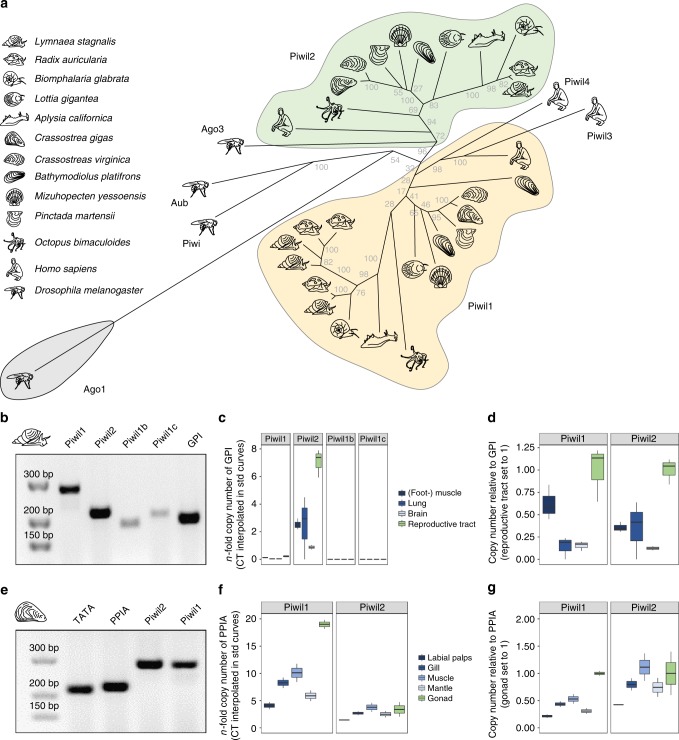
Evolution and expression of *PIWI* genes in mollusks. **a** *PIWI* gene tree reconstruction of molluskan *PIWI* genes. **b** Control PCR with *PIWI* paralog specific primers and *L. stagnalis* cDNA from the reproductive tract. The complete gel is shown in Supplemental Fig. 1g. **c** qPCR results for *PIWI* paralog expression in different tissues of *L. stagnalis*, measured as *n*-fold expression of the housekeeping gene *GPI*. Center line indicates median, box limits represent the 50th percentile, whiskers show the upper and lower extremes. **d** *PIWI* paralog expression in different tissues of *L. stagnalis*, normalized by the expression of the housekeeping gene *GPI*, values from reproductive tract set to 1. Center line indicates median, box limits represent the 50th percentile, whiskers show the upper and lower extremes. **e** Control PCR with *PIWI* paralog specific primers and *C. gigas* cDNA from the adductor muscle. The complete gel is shown in Supplemental Fig. 1h. **f** qPCR results for *PIWI* paralog expression in different tissues of *C. gigas*, measured as *n*-fold expression of the housekeeping gene *PPIA*. Center line indicates median, box limits represent the 50th percentile, whiskers show the upper and lower extremes. **g** *PIWI* paralog expression in different tissues of *C. gigas*, normalized by the expression of the housekeeping gene *PPIA*, values from male gonad set to 1. Center line indicates median, box limits represent the 50th percentile, whiskers show the upper and lower extremes

Many *PIWI* gene tree reconstructions have been published in the past years, however, they do not provide a coherent picture regarding the evolution of *PIWI* genes in early bilaterians. Thus, we first wanted to characterize the PIWI protein equipment of sequenced mollusks to infer the ancestral molluskan state and subsequent evolution of *PIWI* paralogs within the molluskan clade. To this end, we used available PIWI protein sequence data from six molluskan species (*Biomphalaria glabrata*, *Aplysia californica*, *Crassostrea gigas*, *Crassostrea virginica*, *Mizuhopecten yessoensis*, *Octopus bimaculoides*) and further manually annotated *PIWI* genes based on five publicly available but not yet (sufficiently) annotated genomes (*Lymnaea stagnalis*, *Radix auricularia*, *Lottia gigantea*, *Bathymodiolus platifrons*, *Pinctada martensii*). We found that the PIWI family members *Piwil1* and *Piwil2* are conserved in mollusks and are orthologous to *Piwil1* and *Piwil2* in vertebrates, suggesting a duplication event in an early bilaterian ancestor prior to the split of protostomes and deuterostomes. According to our results and in consistency with a number of previously published gene trees, *Drosophila* *AGO3* shares a common ancestral gene with *Piwil2* clade members^18,42–44^. However, the insect-specific *PIWI* genes *Piwi* and *Aubergine*, the latter one resulting from a duplication event in dipteran flies^44,45^, do not group with the *Piwil1* clade (Fig. 1a). It is worth mentioning in this context that different rates of sequence evolution, selective regimes, and gene turnover for Argonaute subfamilies make it difficult to infer their ancient evolutionary history, which is mirrored by numerous published but contradicting *PIWI* gene trees, none of which correctly mirrors the phylogenetic relationship of the included species. Consequently, the presented gene tree reconstruction aims to provide a reliable reconstruction of molluskan *PIWI* gene evolution while the deeper topology should be considered with caution.

While we did not observe further gene duplication events within the molluskan *Piwil2* clade, several duplication events are present in the *Piwil1* clade resulting in two *Piwil1* paralogs in *Bathymodiolus platifrons* and even three *Piwil1* paralogs in *Lymnaea stagnalis* and *Radix auricularia*. Generally, *PIWI* gene duplication events are in line with the previously described erratic evolution of *PIWI* family genes in arthropods^19,44–46^. Noteworthily, it was also a successive duplication of *Piwil1* on the eutherian lineage that gave rise to *Piwil3* (with subsequent loss on the murine lineage) and *Piwil4*^47,48^ (Fig. 1a).

### Expression of *PIWI* genes in *L. stagnalis* and *C. gigas*

To investigate the expression of *PIWI* genes in mollusks we chose two representative species, the pacific oyster *Crassostrea gigas* (*C. gigas*, Bivalvia) showing no *Piwil1* duplication, and the great pond snail *Lymnaea stagnalis* (*L. stagnalis*, Gastropoda), featuring three predicted *Piwil1* paralogs (Fig. 1a). We performed quantitative real-time PCR (qPCR) for each *PIWI* paralog on a representative set of tissues from both species.

For the great pond snail *L. stagnalis* we measured *PIWI* expression on the mRNA level in the hermaphroditic reproductive tract, comprising both male and female gametes, foot muscle, lung, and brain. Relevant expression was detectable for *Piwil1* and particularly *Piwil2*, while the *Piwil1* duplicates *Piwil1b* and *Piwil1c* were only expressed at very low levels (Fig. 1b,c and Supplementary Fig. 1) suggesting a spatiotemporal sub-functionalization. As expected, we observed the highest expression of *Piwil1* and *Piwil2* in the reproductive tract. However, both genes were significantly expressed in the other analyzed tissues as well, reaching 62%, 21%, and 15% of germline expression for *Piwil1* in muscle, lung, and brain respectively, and 36%, 53%, and 12% of germline expression for *Piwil2* in muscle, lung, and brain, respectively (Fig. 1d).

For the dioecious pacific oyster *C. gigas*, *PIWI* mRNA expression was measured in the male gonad, labial palps, gill, adductor muscle, and mantle. We detected significant expression of *Piwil1* and *Piwil2* across all analyzed tissues, particularly in gonadal tissue (Fig. 1e, f), confirming data on *Piwil1* expression in the Hong Kong Oyster *Crassostrea honkongensis*^49^. In relation to gonadal expression, *Piwil1* and *Piwil2* were expressed in levels ranging from 21% (*Piwil1* in labial palps) to 111% (*Piwil2* in adductor muscle, Fig. 1g). The observed expression patterns suggest that a functional PIWI machinery acting in the soma and the germline is conserved in mollusks. Considering the somatic expression of PIWI proteins and piRNAs in many arthropod species^19^, it is parsimonious to assume that somatic PIWI/piRNA expression represents the ancestral state that was established in an early protostomian ancestor.

### piRNAs in *L. stagnalis* muscle and reproductive tract

In order to characterize molluskan piRNAs, we sequenced small RNA transcriptomes from *L. stagnalis* extracted from the hermaphroditic reproductive tract and (foot-) muscle, since muscle tissue was found to exhibit the highest somatic *PIWI* expression in both *L. stagnalis* and *C. gigas*. Importantly, we want to clarify that we will use the term piRNA bona fide, without formal evidence for physical interaction with PIWI proteins but based on the evidence provided in the following.

**Fig. 2:**
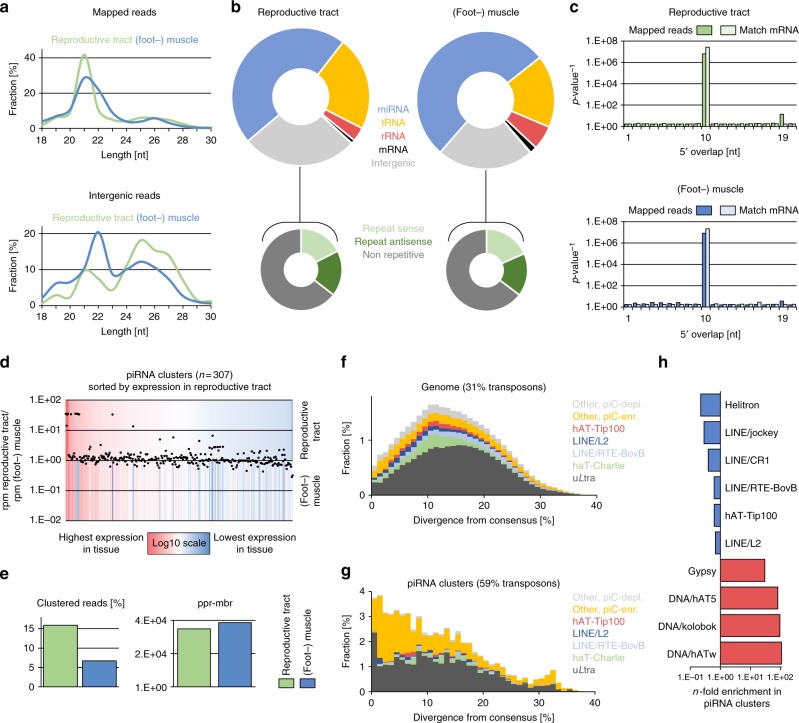
Characterization of small RNAs from *L. stagnalis* (foot-) muscle and reproductive tract. **a** Sequence read length distribution of mapped (top) and unannotated (intergenic) reads (bottom). **b** Results from small RNA annotation with unitas (top) and transposon content of intergenic reads (bottom). **c** Ping-pong signature. *P*-values are deduced from the corresponding *Z*-scores. *P*-values for all reads and reads that match mRNA are shown. **d** Differential expression of 307 predicted piRNA clusters. Colors refer to expression relative to highest/lowest expression within one tissue. Dots indicate *n*-fold expression of a given cluster in reproductive tract relative to muscle. **e** Amount of clustered reads and ping-pong reads per million bootstrapped reads (ppr-mbr). **f** Representation of transposons in the genome of *L. stagnalis*, plotted by divergence [%] from transposon consensus. **g** Representation of transposons within piRNA clusters of *L. stagnalis*, plotted by divergence [%] from transposon consensus. **h** Prominent transposons that are enriched or depleted in *L. stagnalis* piRNA clusters

The sequence read length profiles for both tissues show a maximum for 21 nt RNAs, with a considerable amount of 22 nt RNAs being present in the muscle, but not in the reproductive tract. We further observed a smaller fraction of RNAs in the range of 24–29 nt in both samples (Fig. 2a). Annotation of sRNA sequences with unitas^50^ revealed a similar proportion of different sRNA classes in each tissue type, with miRNAs accounting for 47% and 53% of reads in the reproductive tract and muscle, respectively (Fig. 2b, Supplementary Table 1). Interestingly, we found a substantial difference in the abundance of tRNA fragments (tRFs). In both samples, 21 nt RNAs derived from the 3′ end of tRNAs (3′ tRFs, particularly from tRNA-Gly-TCC) constitute the vast majority of tRNA fragments. However, the share of 3′ tRFs in the reproductive tract is considerably higher compared to muscle (17 and 10%, respectively, Supplementary Table 1). Recently, 3′ tRFs were found to silence long terminal repeat (LTR) retrotransposons in mouse stem cells by targeting their functionally essential and highly conserved primer-binding sites^51^. The remarkable amount of 3′ tRFs in the analyzed samples supports the idea proposed by Schorn et al. ^51^ who assume that this mechanism could be highly conserved across different species, providing an innate immunity against LTR propagation.

Focusing on putative piRNAs, we analyzed the fraction of sequence reads that did not match to any other class of non-coding RNA nor mRNA. This dark matter of intergenic sRNAs comprises 27% and 23% of sequence reads in the reproductive tract and in muscle, respectively, and is enriched for transposon sequences, suggesting a role in transposon control (Fig. 2b). Analyses of their sequence read length distribution revealed a prominent class of 22 nt molecules in muscle and to a lesser extend in the reproductive tract, suggesting that transposon defense in *L. stagnalis* involves 22 nt siRNAs in addition to piRNAs (Fig. 2a). To verify the presence of piRNAs, we checked for the so-called ping-pong signature (bias for 10 bp 5′ overlap of mapped sequence reads), which is a hallmark of secondary piRNA biogenesis and requires the catalytic activity, and thus expression, of PIWI proteins^52^. Remarkably, we detected a significant ping-pong signature in both, the reproductive tract and muscle (Fig. 2c), suggesting active PIWI/piRNA-dependent transposon silencing in the germline and in the soma. In addition, a ping-pong signature can also be observed for sequence reads that match protein-coding genes, indicating piRNA-dependent gene regulation (Fig. 2c).

Next, we used proTRAC^53^ to identify 308 piRNA-producing loci in the reproductive tract, and 246 piRNA-producing loci in muscle tissue. Merging of independently annotated contiguous (<10 kb distance) or overlapping piRNA-producing loci revealed a total of 307 distinct piRNA clusters in *L. stagnalis*, covering 0.27% of the genome (Fig. 2d, Supplementary Data 1). More precisely, all piRNA-producing loci identified in muscle tissue correspond to predicted piRNA clusters based on piRNAs from the reproductive tract, which illustrates that piRNAs in muscle originate from the same set of piRNA clusters compared to the reproductive tract. Nonetheless, there exist 12 clusters whose expression is 14-fold to 36-fold higher in the reproductive tract compared to muscle tissue, while no clusters show muscle-specific expression to a comparable extent. We found that 15.9% of sequence reads from the reproductive tract map to piRNA clusters, while only 6.7% of sequence reads from muscle do so, indicating rather moderate production of primary piRNAs in the soma compared to the germline (Fig. 2e). Besides the presence of primary piRNAs, we found that the number of piRNAs that participate in ping-pong amplification (measured as ping-pong reads per million bootstrapped reads, ppr-mbr) is slightly higher in muscle (~39k ppr-mbr) compared to the situation in the reproductive tract (~35k ppr-mbr), suggesting higher amounts of secondary piRNAs and emphasizing the functional importance of somatic PIWI/piRNA expression (Fig. 2e). In line with the transposon-suppressive role of piRNAs, the identified piRNA clusters show a twofold enrichment for transposon sequences compared to the whole genome situation (59 and 31%, respectively, Fig. 2f, g), whereas only 1.7% of piRNA cluster sequence represents protein-coding sequence. Interestingly, the transposon composition in piRNA clusters does not at all reflect the transposon landscape of the genome. Instead, piRNA clusters are enriched for Gypsy retrotransposons and particularly DNA transposons such as Kolobok, hAT5, or hATw showing up to 108-fold enrichment in piRNA clusters (Fig. 2g, h). This non-random distribution suggests a selective regime that favors insertion events of transposons with low divergence from their consensus sequence, likely representing evolutionary young and active elements.

### Ubiquitous and dynamic expression of piRNAs in *C. gigas*

**Fig. 3:**
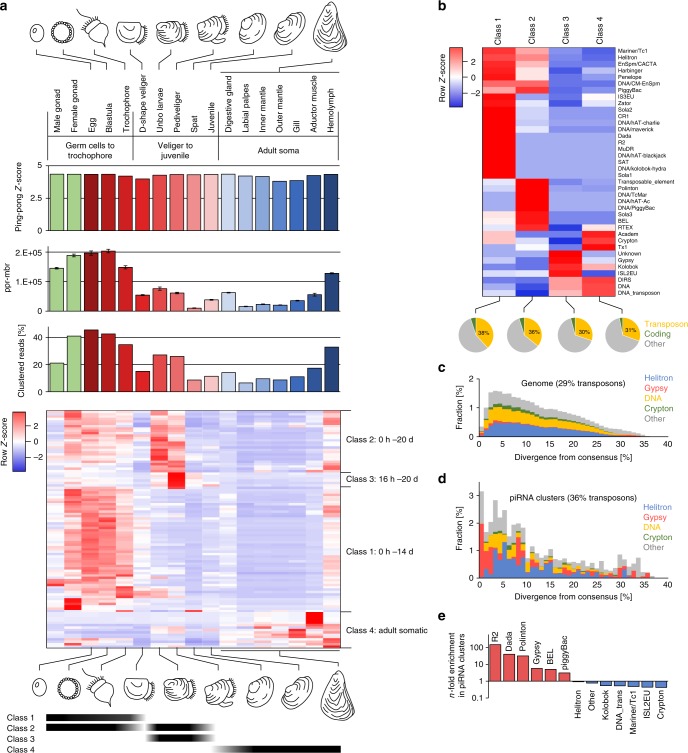
Characterization of small RNAs and piRNA clusters from different *C. gigas* samples. **a** Sequence reads without annotation produce a significant ping-pong signature (top row of bars, only *Z*-scores for 10 bp 5′ overlap are shown). The number of ping-pong reads per million bootstrapped reads (middle row of bars), and the number of clustered reads (bottom row of bars) differs considerably across the samples. Heatmap shows the differential expression of the top 100 piRNA clusters in terms of maximum rpm coverage. Different classes of piRNA clusters are expressed during oyster development and in adult somatic tissues (bottom). Error bars indicate standard deviation. **b** Transposon composition of piRNA clusters belonging to four different classes. **c** Representation of transposons in the genome of *C. gigas*, plotted by divergence [%] from transposon consensus. **d** Representation of transposons within piRNA clusters of *C. gigas*, plotted by divergence [%] from transposon consensus. **e** Prominent transposons that are enriched or depleted in *C. gigas* piRNA clusters

Based on our observation that *PIWI* genes and piRNAs are expressed in the soma and the germline of *L. stagnalis*, we reanalyzed previously published small RNA data sets from *C. gigas* that were used to investigate the dynamic expression of miRNAs during oyster development without further examination of a putative piRNA fraction^54^ (NCBI Sequence Read Archive Project ID SRP007591). We annotated *C. gigas* sRNAs from the male and female gonad, different developmental stages ranging from the egg to juvenile, and a representative set of somatic tissues from adult animals (Supplementary Table 2). In all data sets, particularly in gonadal tissues, eggs, and early embryo stages but also in hemolymph we detected a large amount of sequence reads that did not match to any known ncRNA class but was instead enriched for transposon sequences. The transposon-matching sub-fraction itself was enriched for antisense sequences (Supplementary Table 2). Analogous to the procedure applied for the *L. stagnalis* data sets, we verified the presence of primary and secondary piRNAs by analyzing the ping-pong signature of each data set. Remarkably, we detected a significant ping-pong signature across all analyzed data sets (Fig. 3a, Supplementary Fig. 2), but also found that the number of ping-pong reads (measured as ppr-mbr) differs considerably depending on the tissue and developmental stage (Fig. 3a, Supplementary Fig. 3). Noteworthily, as is the case with *L. stagnalis*, a ping-pong signature is also detectable when taking only those reads into account that match protein-coding sequences, suggesting a relevant and conserved role of the PIWI/piRNA pathway in post-transcriptional regulation of protein-coding genes in gonads, egg, blastula, digestive gland, and hemolymph (Supplementary Table 3). We further used sequences without ncRNA annotation to predict piRNA clusters with proTRAC (Supplementary Data 2) and checked whether we can observe a differential expression of specific piRNA clusters in time and space (Fig. 3a).

In contrast to the situation in *L. stagnalis*, we found that different genomic loci are responsible for production of primary piRNAs in the germline and in the soma, but also during different developmental stages, which is similar to the situation in the sea anemone *Nematostella vectensis*^18^ and the German cockroach *Blatella germanica*^55^. A clustering approach based on average linkage^56^ revealed four distinct groups of piRNA clusters which we named class 1–4 piRNA clusters (Fig. 3a). Class 1 piRNA clusters are active in the adult germline (male and female) and in the early embryo until the D-shaped veliger stage where larvae are ~14 h old. The same applies to class 2 piRNA clusters, however, following the D-shape veliger stage, class 1 piRNA clusters become inactive, while class 2 piRNA clusters remain active and class 3 piRNA clusters start piRNA production. Both, class 2 and class 3 piRNA cluster activity is measurable until the juvenile stage, where oysters are ~20 days old. In somatic tissues of adult oysters, class 4 piRNA clusters represent the main source of primary piRNAs (Fig. 3a, bottom). Interestingly, all four classes of piRNA clusters are active in hemocytes, which also feature the highest amount of clustered reads, and ping-pong reads compared to other somatic tissues. This might reflect the presence of stem cells within the hemocyte cell population, which are subject to complex differentiation processes^57,58^.

Interestingly, the four classes of piRNA clusters differ considerably regarding the overall transposon content as well as the specific transposon composition (Fig. 3b–d). Class 1 and class 2 piRNA clusters are generally enriched for transposon sequences showing 38 and 36% transposon-derived sequences, respectively, compared to a genomic transposon content of 29%. The surprisingly high accumulation of young (as deduced from the divergence from their consensus) Gypsy elements in piRNA clusters, suggests a strong selection for Gypsy element insertions, probably as a consequence of Gypsy activity in *C. gigas*. Noteworthily, the accumulation of young transposons in molluskan piRNA clusters sharply contrasts the situation in *Drosophila* and human, where older transposons are more abundant in piRNA-producing loci^59,60^. Considering transposons that are generally enriched in piRNA clusters, we found that R2 retrotransposons (149-fold enrichment in piRNA clusters) and Dada DNA transposons (40-fold enrichment in piRNA clusters) are most abundant in class 1 piRNA clusters (Fig. 3e). In contrast, Polinton DNA transposons (32-fold enrichment in piRNA clusters) and BEL retrotransposons (fivefold enrichment in piRNA clusters) are most abundant in class 2 piRNA clusters. Different from class 1 and class 2 piRNA clusters, class 3, and class 4 piRNA clusters display only slight transposon enrichment (30 and 31%, respectively). Noteworthily, high copy number Gypsy retrotransposons (fivefold enrichment in piRNA clusters) are most abundant in class 3 piRNA clusters, while Academ, Crypton, and Tx1 transposons are most abundant in class 4 piRNA clusters.

The fact that different piRNA clusters are expressed in the germline (class 1 and class 2) and in adult somatic tissues (class 4) of *C. gigas* contrasts with the situation in *L. stagnalis*, where identical piRNA-producing loci are active in the germline and in the soma. Moreover, we can observe considerable differences in the transposon composition of piRNA clusters in the two species, which likely reflect a divergent transposon activity in gastropods and bivalves, resulting in varying selective constraints on the different phylogenetic lineages.

### Homotypic and heterotypic ping-pong amplification

The ping-pong amplification loop describes a process that is responsible for the post-transcriptional silencing of transposable elements^52^. In *Drosophila* and mouse, this process typically involves two PIWI paralogs (heterotypic ping-pong), one loaded with antisense piRNAs targeting transposon transcripts, and the other loaded with sense piRNAs targeting piRNA cluster transcripts, which contain transposon sequences in antisense orientation^61,62^. Likely for steric reasons, premature piRNAs loaded onto the different PIWI paralogs are more or less rigorously trimmed at their 3′ ends. This is why piRNA populations bound to different PIWI paralogs not only differ regarding the amount of sense-transposon and antisense-transposon sequences, but also in their sequence length profiles^52,63,64^. In addition to the heterotypic ping-pong amplification, homotypic ping-pong has been shown to occur in *qin* mutant flies (Aub:Aub^65^), and wild-type prenatal mouse testis (Miwi2:Miwi2, Mili:Mili^62^).

**Fig. 4:**
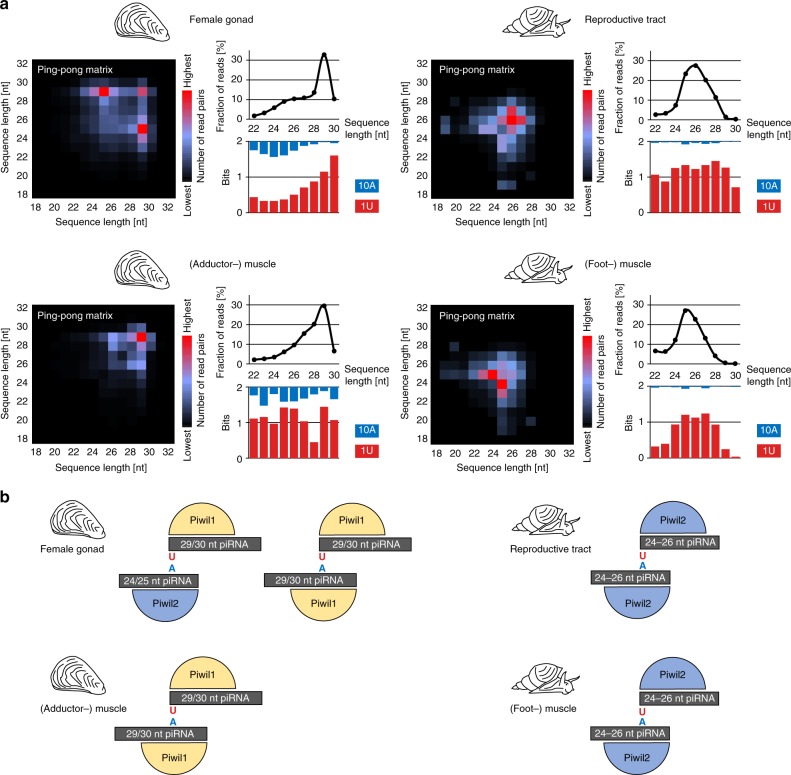
Analysis of piRNAs that participate in the ping-pong amplification loop. **a** Ping-pong matrices illustrate frequent length-combinations of ping-pong pairs (sequences with 10 bp 5′ overlap). Sequence read length distribution and 1U/10A bias [bits] for ping-pong sequences are shown. **b** Proposed model of ping-pong amplification in the germline and muscle of *C. gigas* and *L. stagnalis*

Since the typical molluskan genome encodes two ubiquitously expressed PIWI paralogs, *Piwil1* and *Piwil2*, we asked whether we can provide evidence for the participation of distinct piRNA populations and PIWI paralogs in the ping-pong cycle. We conducted a bioinformatics approach under the premise that Piwil1-bound and Piwil2-bound piRNAs exhibit different length profiles, which is the case for the corresponding mouse homologs Piwil1 (Miwi) that preferentially binds 29/30 nt piRNAs, and Piwil2 (Mili) which preferentially binds 26/27 nt piRNAs^66^. A similar, yet not equally pronounced, difference between Piwil1-bound (Ziwi) and Piwil2-bound (Zili) piRNAs also exists in zebrafish, suggesting the evolutionary conservation of this pattern^8^. We analyzed pairs of mapped *C. giga*s and *L. stagnalis* sequence reads that showed a 10 bp 5′ overlap (ping-pong pairs), with respect to the sequence length of each ping-pong partner (Fig. 4, Supplementary Fig. 4). In the female gonad of *C. gigas*, most ping-pong pairs combine piRNAs with a length of 25 nt and 29 nt (Fig. 4a), suggesting heterotypic Piwil1–Piwil2-dependent ping-pong amplification as depicted in Fig. 4b. In support of this, 29 nt piRNAs, presumably bound to Piwil1, are heavily biased for a 5′ uridine (a hallmark of primary piRNAs), whereas 25 nt piRNAs, presumably bound to Piwil2, show a stronger bias for an adenine at position 10 (typical for secondary piRNAs). In contrast, ping-pong pairs in *C. gigas* muscle predominantly combine two 29 nt piRNAs, suggesting homotypic, Piwil1-dependent ping-pong amplification (Fig. 4b). Generally, the observed patterns of ping-pong pairs are very diverse across the different samples, for instance displaying heterotypic ping-pong in the digestive gland and homotypic Piwil2-dependent ping-pong in hemolymph cells (Supplementary Fig. 4).

Since the expression of *Piwil1* compared to *Piwil2* is considerably lower in *L. stagnalis*, we were curious to check whether the corresponding ping-pong pairs might reflect this fact. Indeed, 26/26 nt pairs (homotypic, Piwil2-dependent ping-pong) represent the majority of ping-pong pairs in the reproductive tract (Fig. 4a). In addition, homotypic Piwil2-dependent ping-pong amplification with 24/25 nt ping-pong pairs is also dominant in the *L. stagnalis* muscle (Fig. 4b). However, we also observed differences in ping-pong patterns that do not correlate with the measured mRNA levels of *Piwil1* and *Piwil2*. For example, our data suggests homotypic Piwil2-dependent ping-pong amplification in the oyster gill but homotypic Piwil1-dependent ping-pong amplification in the oyster muscle (Supplementary Fig. 4), while both tissues display a very similar expression of both *PIWI* paralogs on the mRNA level (Fig. 1f). Thus, we assume that factors other than mere PIWI expression critically influence characteristics of the ping-pong amplification loop.

Moreover, we clearly cannot rule out the possibility that binding preferences of PIWI paralogs have changed on the molluskan lineage and are different from those observed in fly, fish, and mouse. This could mean that length profiles of piRNAs associated to each of the molluskan PIWI paralogs might be exactly reciprocal compared to our presumption. One could even speculate that both PIWI paralogs may bind the whole range of piRNAs, which is not possible to disprove without performing corresponding co-immunoprecipitation experiments. However, based on the presence of piRNA populations with different length profiles (Fig. 2a), their representation in ping-pong pairs together with the differences in their amount of 1U and 10A reads (Fig. 4a), we believe that the above made interpretations are a reasonable and parsimonious interpretation of the data at hand, yet not the only possible one.

## Discussion

Our results reveal that mollusks utilize the PIWI/piRNA pathway as a defense against transposable elements in the germline and in the soma, which corresponds to the situation in arthropods and therefore suggests somatic PIWI/piRNA expression to represent a plesiomorphic protostomian character state. In fact, available data from deeper branching metazoans such as poriferans and cnidarians supports the view that this system was established in the soma even long before the split of protostomes and deuterostomes^7,18,41^. In addition, based on the observation that a substantial fraction of arthropod and mollusk piRNAs targets messenger RNAs producing the generic ping-pong signature, it seems likely that the last common ancestor of arthropods and mollusks applied the PIWI/piRNA pathway also for post-transcriptional regulation of protein-coding genes. Recently, the Xenacoelomorpha phylum, a group of marine worms that were previously thought to belong to the Platyhelminthes clade, was found to represent the sister group of Nephrozoa which comprise protostomes and deuterostomes^67,68^. Presently, piRNAs for this outgroup are not characterized but having such data would doubtlessly provide valuable insights and allow to draw conclusions regarding the function of the PIWI/piRNA system in the last common ancestor of all bilaterians, particularly with respect to an ancestral gene-regulatory role. Especially with regard to the latter, functional studies in non-model organisms are urgently needed since the pure bioinformatical evidence for piRNA-dependent processing of protein-coding genes does not give any information on its factual biological relevance this process might have in different species. In vertebrates, somatic PIWI/piRNA expression appears to have faded away and reports on somatically expressed piRNAs in mammals are often considered with skepticism for good reasons^69^. However, remnants of the former somatic expression might have outlasted to fulfill special functions in specific cells and/or in narrowly defined timespans of development or cell differentiation in the one or the other clade. Our results indicate that studying the PIWI/piRNA pathway in organisms outside of the main experimental models of *Drosophila* and mouse is necessary to fully understand its evolution and functions.

## Methods

### *PIWI* gene annotation and tree reconstruction

In order to reconstruct the phylogenetic relations of mollusk Piwi proteins, we first searched for *PIWI* genes in species with an available genome sequence that lack proper annotation (*Lymnaea stagnalis*, *Radix auricularia*, *Lottia gigantea*, *Bathymodiolus platifrons*, *Pinctada martensii*). To this end, we scanned the relevant genomes for sequences that are homologous to annotated *PIWI* paralogs of the pacific oyster (EKC35279 and EKC29295) by aligning translated DNA sequences using tblastx (v2.7.1+ ^70^,). Neighboring hits with a distance smaller than 10 kb were grouped as exons of distinct gene loci. Only groups containing the overall best hits for a given locus were retained. Finally, the predicted gene sequences were checked for presence of PIWI and PAZ domains using NCBI conserved domain database^71^. Similarly, for *PIWI* expression analysis by qPCR in the pond snail, we identified the housekeeping gene *GPI* (glucose-6-phosphate isomerase) by comparison with the human ortholog (ARJ36701).

The predicted and annotated PIWI protein sequences of the 11 available molluskan species together with PIWI paralogs of human (Piwil1–4) and fly (Ago3, Piwi, Aub), as well as fly argonaute Ago1 were aligned using MUSCLE (v.3.8.31^72^,). Subsequently, the resulting protein alignment was curated with Gblocks (v.0.91b), allowing smaller final blocks with gap positions and less strict flanking positions. Using ModelGenerator (v.0.85^73^,) we determined LG + G + F^74^ to be the best-fitting model of substitution for our data. The curated alignment (Supplementary Data 3) was then used for phylogenetic tree reconstruction with PhyML (v3.1^75^,) applying approximate likelihood-ratio test (SH-like) and LG substitution model, including empirical gamma distribution (G) and character frequencies (F). Support values were generated by bootstrap with 100 replicates.

### qPCR

Experiments were performed on commercially available *C. gigas* animals from the western French Atlantic coast (lle d’Oleron) and captured wild living *L. stagnalis* animals from South-western Germany (Heppenheim). To estimate the expression of the *Piwil* homologs in several tissues of *L. stagnalis* and *C. gigas* we performed qPCR with cDNA synthesized from the total RNA fraction of these tissues. Total RNA was isolated with TriReagent and the polyadenylated transcriptome was reversely transcribed with SuperScript IV using the RT-primer 5′-CGAATTCTAGAGCTCGAGGCAGGCGACATGT_25_VN-3′. Primers amplifying ~200 bp long products of the respective *Piwil* homologs and housekeeping genes were designed with the NCBI tool primer-BLAST on basis of the *L. stagnalis* genome assembly GCA_900036025.1 v1.0 and the *C. gigas* genome assembly GCA_000297895.1 oyster_v9. To prevent amplification of residual genomic DNA, primers were designed to be exon-junction spanning or to span at least several intronic regions. The respective biological replicates were analyzed as technical duplicates on a Corbett Rotor-Gene 6000 real-time PCR cycler and the copy numbers of the genes of interest were quantified by standard curves of the individual primer pair amplicons. For each cDNA sample the calculated *Piwil* copy numbers were relativized by the calculated copy numbers of the housekeeping genes to calibrate for variabilities in sample preparation. These *n*-fold expression values were finally used to calculate the mean and standard deviation of the replicates. For an improved visualization, the *n*-fold expression values of each *Piwi* homolog are additionally displayed as a percentage of the respective gonad value.

### Small RNA extraction and sequencing

We extracted total RNA from *L. stagnalis* reproductive tract (incl. ovotestis, oviduct, spermatheca, spermiduct, prostate, uterus, vagina, vas deferens) and foot muscle, and total RNA from *C. gigas* adductor muscle and gonadal tissue with TriReagent according to the manufacturerʼs instructions. For each species we sampled two different individuals per tissue. The small RNA fractions of each obtained total RNA sample were sequenced at BGI, Hong Kong, on a BGISEQ-500 unit. Small RNA sequence data sets for *L. stagnalis* and *C. gigas* are deposited at NCBIʼs Sequence Read Archive (SRA) and can be accessed under the SRA project IDs SRP130729 and SRP130745. We further used previously published small RNA sequence data from *C. gigas*^54^ to analyze piRNA expression and characteristics with respect to different developmental stages.

### Repeat annotation

We performed de novo prediction of repetitive elements in the genome of *L. stagnalis* with RepeatScout (v. 1.0.5^76^). Predicted repetitive elements were classified with RepeatClassifier which is part of the RepeatModeler (v. 1.0.11) package. Transposons that failed to be classified based on known transposons from other species are referred to as unclassified *Lymnaea*-specific transposons (u*L*tra). The resulting repeat sequences, as well as a complete collection of currently available molluskan repeat sequences from RepBase^77^ were used as reference sequences for repeat masking of the *L. stagnalis* and *C. gigas* genomes with RepeatMasker (v. 4.0.7) using the cross_match search engine and the option -s for most sensitive masking. Annotated repeats in the RepeatMasker output were analyzed with respect to transposon families and divergence from their consensus sequence using the Perl script TE_landscape.pl. Analysis was conducted with the entire repeat data set as well as with repeats localized in predicted piRNA clusters. TE_landscape.pl is freely available at https://sourceforge.net/projects/protrac/files/tools/.

### Gene annotation

We performed de novo gene annotation of the *L. stagnalis* genome assembly gLs_1.0^78^ using the MAKER genome annotation pipeline (v.2.31.8) in order to identify sRNAs that match protein-coding sequences^79^. Initially, we masked the *L. stagnalis* genome with WindowMasker^80^ using default settings including the duster option to mask low-complexity regions. Then, we used available molluskan cDNA data from Ensembl database (release 92) and available mRNA and protein data from *L. stagnalis* deposited at NCBI (Effective April 25, 2018) as input for MAKER. MAKER output files for separate scaffolds were merged using the Perl script mergeMAKERoutput.pl which is freely available at https://sourceforge.net/projects/protrac/files/tools/. The complete genome annotation in GFF3 format and a corresponding mRNA sequence file in FASTA format are available as Supplementary Data 4 and Supplementary Data 5.

### Processing and annotation of small RNA sequence data

Small RNA sequence data sets were collapsed to non-identical sequences, retaining information on sequence read counts using the Perl script *collapse*. Sequences > 36nt were rejected using the Perl script *length-filter*. Finally, low-complexity sequences were filtered using the Perl script *duster* with default parameters. All Perl scripts mentioned are part of the NGS toolbox^81^.

We then applied a customized mapping strategy of the remaining small RNA sequence reads based on the consideration that our data sets presumably contain considerable amounts of transposon-derived piRNAs as well as post-transcriptionally edited (e.g., A-to-I) or tailed miRNAs and piRNAs. Genomic mapping was performed with SeqMap^82^ using the option/output_all_matches and allowing up to three mismatches. The obtained alignments were further filtered using the Perl script seqmap_filter.pl that is freely available at https://sourceforge.net/projects/protrac/files/tools/. For the final alignments we allowed up to two non-template 3′ nucleotides and up to one internal mismatch. For each sequence, we only considered the best alignments in terms of mismatch counts, but did not reject alignments with equal quality in case of multiple mapping sequences. Sequences that did not produce at least one valid alignment to the reference genome were rejected.

To improve small RNA sequence annotation, we performed de novo tRNA, rRNA, and miRNA prediction based on the available reference genome assemblies gLs_1.0 (*L. stagnalis*) and GCA_000297895.1 oyster_v9 (*C. gigas*). tRNA annotation was performed with a local copy of tRNAscan (v.1.3.1^83^). Only tRNAs with <5% N’s were taken for further analysis. rRNA sequences were predicted using a local copy of RNAmmer (v.1.2^84^) and hmmer (v.2.2 g^85^). Both tools were run with default parameters. We pooled small RNA sequence reads from different replicates and tissues for each species separately to perform miRNA *de novo* prediction with ShortStack (v.3.8.4^86^) using default parameters. The predicted tRNA, rRNA, and miRNA precursor sequences, as well as previously published miRNA precursor sequences^54,87,88^, were used as additional reference sequences for small non-coding RNA annotation with unitas (v.1.4.6^50^) which was run with the option -riborase. For *L. stagnalis*, we also included predicted cDNA data based on MAKER annotation (see above). sRNA sequences that did not match to any ncRNA or mRNA of *C. gigas* or *L. stagnalis* were blasted against NCBI nucleotide collection (nr) to search for possible contaminants of parasitic species. Sequences that produced better alignments to genomes of species that possibly parasitized the sampled individuals (*Dicrocoelium*, *Legionella*, *Panagrellus*, *Thelazia, Trichobilharzia*) were considered as contaminants and not used for downstream analyses.

### piRNA cluster identification

Sequences that did not produce a match to known non-coding RNAs were considered as putative piRNAs and were used for prediction of piRNA clusters with proTRAC (v. 2.4.0^53^) applying default settings. piRNA clusters were predicted for each data set and species separately. The resulting piRNA cluster predictions for each species were condensed, merging clusters with <10 kb distance from each other using the Perl script merge_clusters which is freely available at https://sourceforge.net/projects/protrac/files/tools/. To preclude false positive annotation of, e.g., tRNA or rRNA genes as piRNA clusters, we validated predicted piRNA clusters by analyzing sRNA reads that mapped to them with respect to their relation to mRNA or other ncRNA classes (Supplementary Fig. 5a). To further check whether piRNA cluster calling may underestimate or overestimate the number of primary piRNAs in our data sets, we performed an arithmetical approach to estimate the fraction of genuine primary piRNAs based on the fraction of 5′ U reads in annotated and non-annotated reads with 24–29 nt length which yields results very close to the number of clustered reads (Supplementary Methods, Supplementary Fig. 5b). We calculated the sequence read coverage (rpm) for each of the resulting piRNA clusters per data set. For *C. gigas* piRNA clusters, a heat map for the top 100 piRNA clusters in terms of maximum rpm coverage (accounting for 64% of summed rpm values) was constructed with Heatmapper^56^ applying Pearson distance and average linkage clustering. Finally, predicted piRNA clusters were analyzed with respect to their repeat and gene content using the Perl script piC_content.pl which is freely available at https://sourceforge.net/projects/protrac/files/tools/.

### Ping-pong quantification

In order to compare ping-pong signatures across multiple data sets with different sequencing depth, we constructed a software tool, PPmeter (v.0.4), that creates bootstrap pseudo-replicates from original data sets and subsequently analyzes the ping-pong signature and number of ping-pong sequence reads of each pseudo-replicate (default: 100 pseudo-replicates each comprising one million sequence reads). The obtained parameters ‘ping-pong score per million bootstrapped reads’ (pps-mbr) and ‘ping-pong reads per million bootstrapped reads’ (ppr-mbr) can be used for quantification and direct comparison of ping-pong activity in different small RNA data sets. The software is freely available at http://www.smallRNAgroup.uni-mainz.de/software.html and https://sourceforge.net/projects/protrac/files/tools/.

### Code availability

Source code of software that has been written for data processing and analysis is freely available at https://sourceforge.net/projects/protrac/files/tools/.

## Electronic supplementary material

Supplementary Information

Supplementary Data 1

Supplementary Data 2

Supplementary Data 3

Supplementary Data 4

Supplementary Data 5

Description of additional supplementary items

## Data Availability

Sequence data have been uploaded to NCBI’s Sequence Read Archive and can be accessed via the accessions SRP130729 and SRP130745.
