# Supplementary material for: PIWI genes and piRNAs are ubiquitously expressed in mollusks and show patterns of lineage-specific adaptation
Source: Commun Biol. 2018 Sep 7;1:137. doi: 10.1038/s42003-018-0141-4 (PMC6128900; doi:10.1038/s42003-018-0141-4)
Supplement: Supplementary file 1 — Supplementary Information [file 42003_2018_141_MOESM1_ESM.pdf]

Supplementary Figure 1

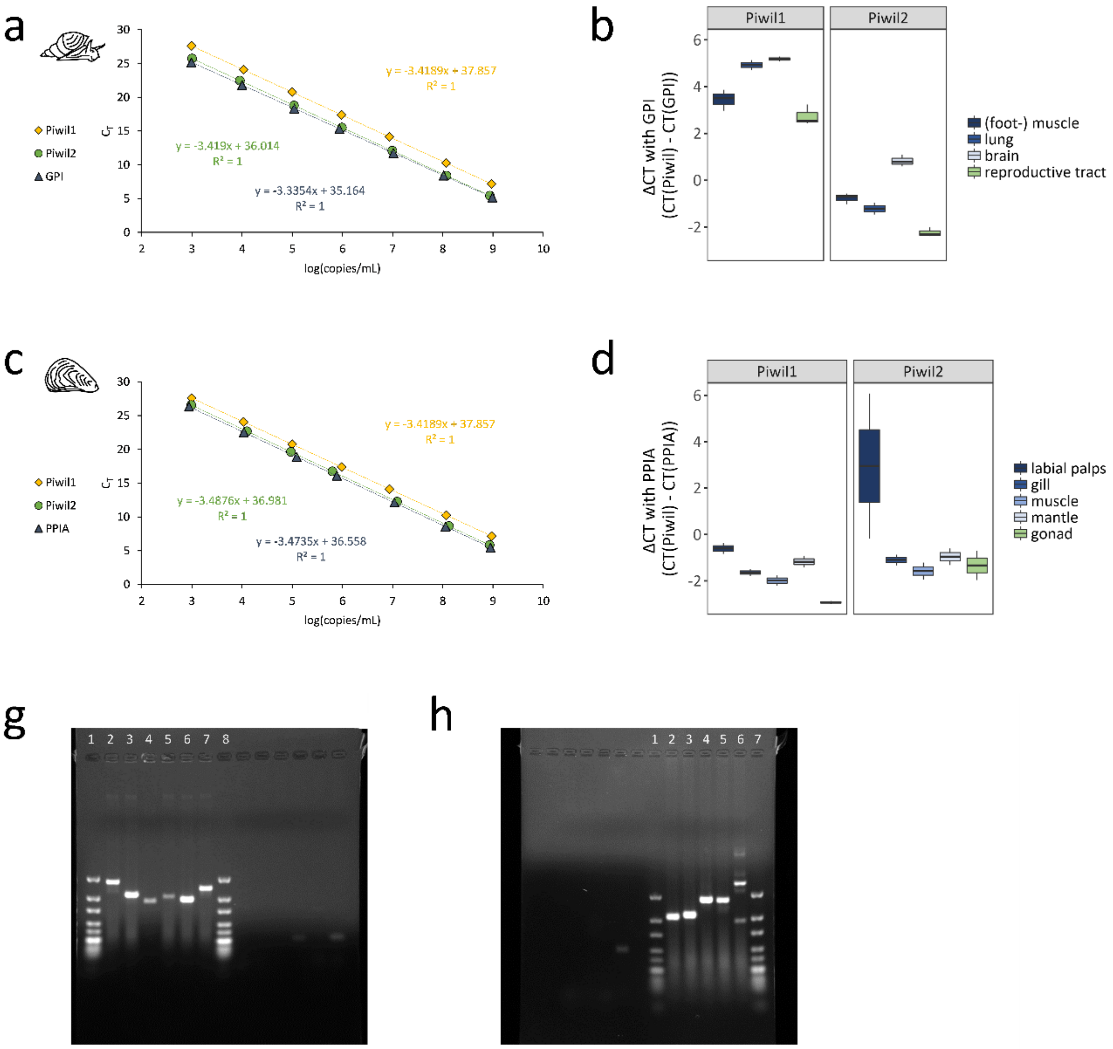

RT-qPCR quantification of PIWI paralog expression using the standard curve method and the ΔC_q method. (a) Standard curves used for absolute quantification of the PIWI homolog transcripts and the transcripts of the housekeeping gene glucose phosphate isomerase (GPI) in different tissues of *L. stagnalis*. The GPI copy numbers of each sample were used to calibrate the copy numbers of the PIWI paralogs for variabilities in sample preparation as shown in Figure 1c and 1d. (b) Relative expression of the PIWI paralog transcripts in *L. stagnalis* as calculated by the ΔC_q method, where $\Delta C_q = \Delta C_q$ (PIWI paralog) - ΔC_q (GPI). Higher ΔC_q values represent lower PIWI expression. (c) Relative expression of the PIWI paralog transcripts in *L. stagnalis* as determined by the ΔCq method. (d) Standard curves used for absolute quantification of the PIWI homolog transcripts and the transcripts of the housekeeping gene peptidylprolyl isomerase A (PPIA) in different tissues of *C. gigas*. The PPIA copy numbers of each sample were used to calibrate the copy numbers of the PIWI paralogs for variabilities in sample preparation as shown in Figure 1f and 1g. (e) Relative expression of the PIWI paralog transcripts in *C. gigas* as calculated by the ΔC_q method, where $\Delta C_q = \Delta C_q$ (PIWI paralog) - ΔC_q (PPIA). Higher ΔC_q values represent lower PIWI expression. Error bars indicate standard deviation. (f) Relative expression of the PIWI paralog transcripts in *C. gigas* as determined by the ΔCq method. (g) Control PCR with PIWI paralog specific primers and *L. stagnalis* cDNA from the reproductive tract. Complete gel from figure 1b. Probes in lanes from 1-8 are: Ultra Low Range DNA Ladder (Thermo Scientific), Piwil1 amplicon, Piwil2 amplicon, Piwil1b amplicon, Piwil1c amplicon, GPI amplicon, EMC7 amplicon, Ultra Low Range DNA Ladder. (h) Control PCR with PIWI paralog specific primers and *C. gigas* cDNA from adductor muscle. Complete gel from figure 1e. Probes in lanes from 1-7 are: Ultra Low Range DNA Ladder (Thermo Scientific), Piwil1 amplicon (primers target both annotated Piwil1 splice isoforms), Piwil1 amplicon (primers target the 18-exon Piwil1 splice isoforms), Piwil2b amplicon, PPIA amplicon, TATA amplicon, Ultra Low Range DNA Ladder.

Supplementary Figure 2

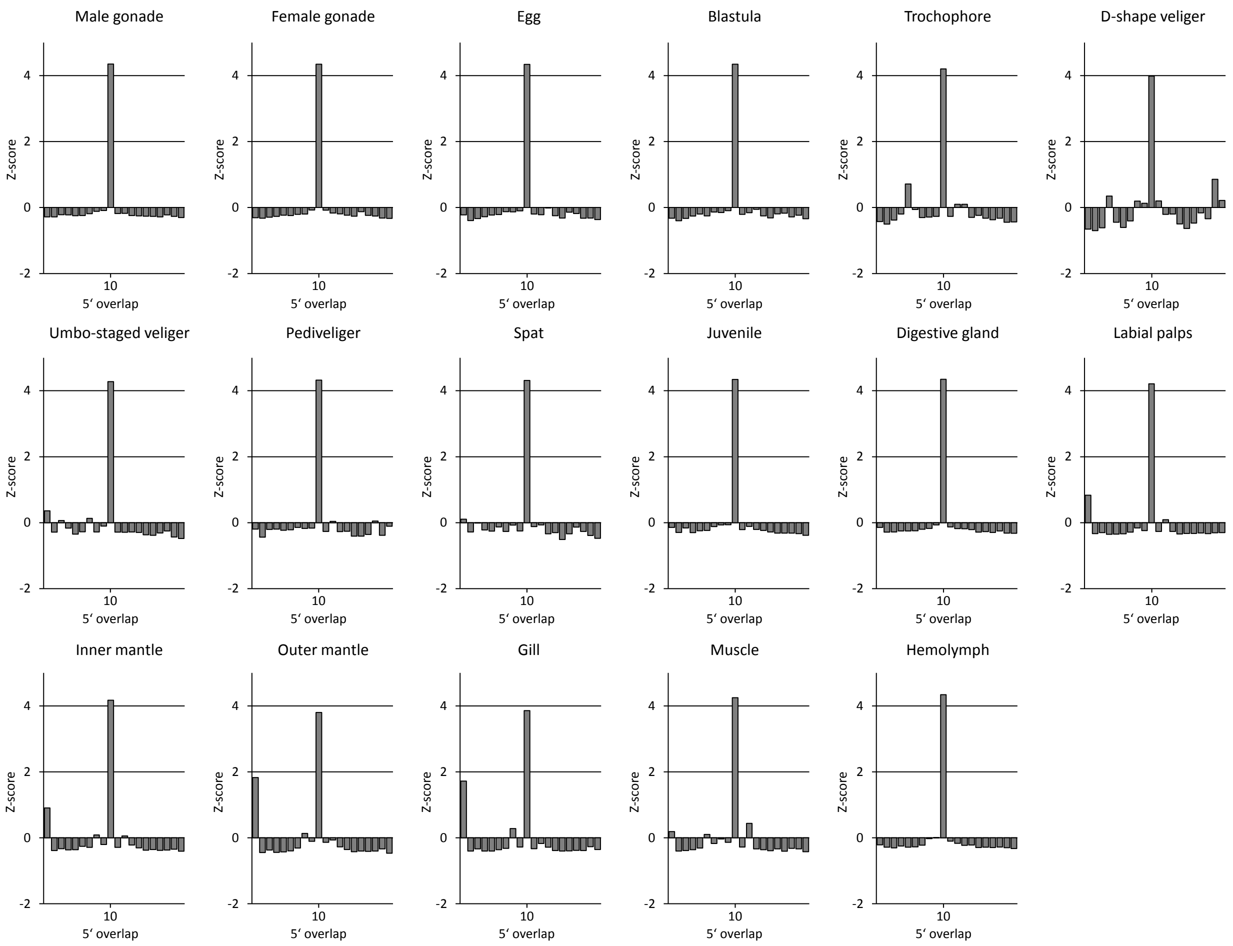

Ping-pong signature of small RNAs from different *C. gigas* samples. Z-scores for specific 5’ overlaps for each sample are shown.

Supplementary Figure 3

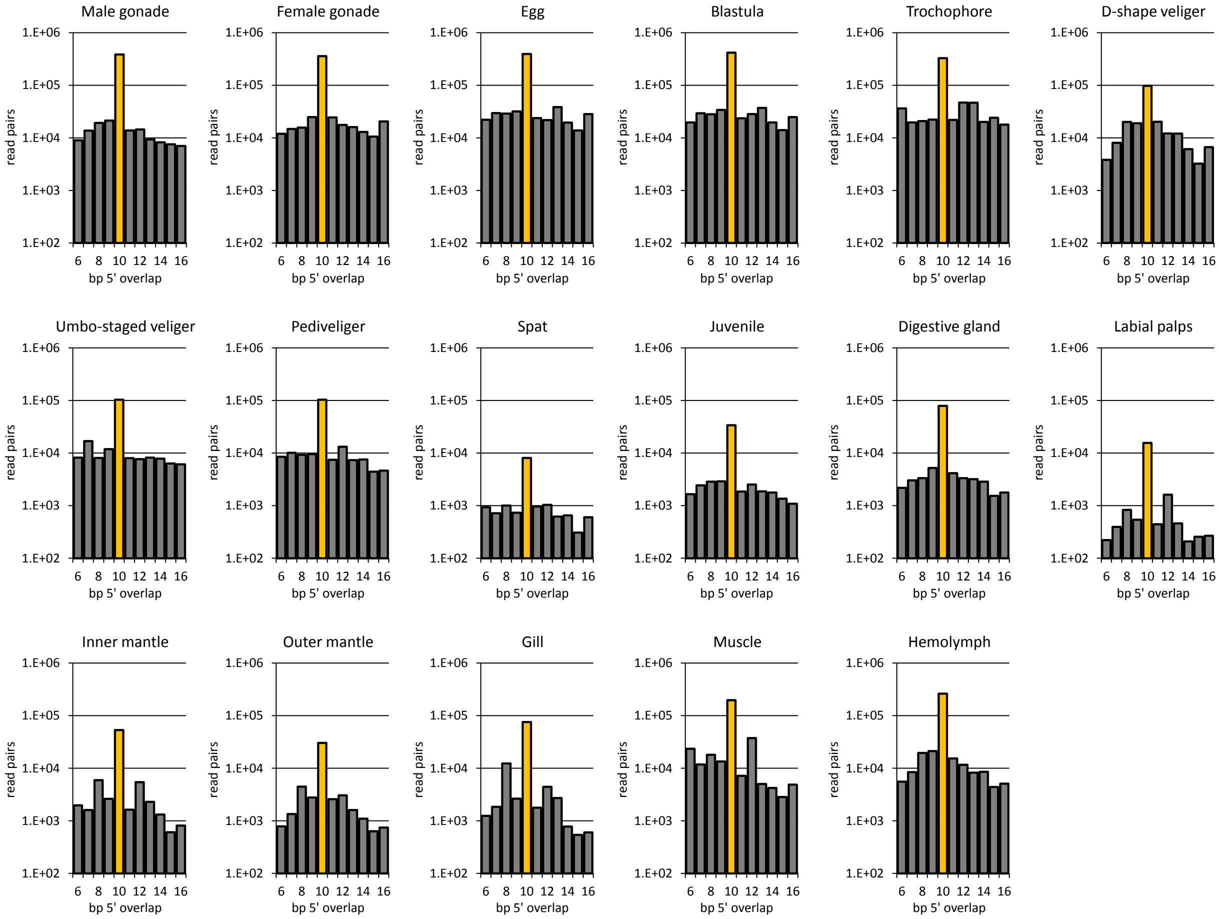

Ping-pong read pairs per million bootstrapped reads from different *C. gigas* samples. Graphs depict the average number of sequence read pairs with a specific 5‘ overlap for 100 pseudo-replicates (PR) per dataset with one million reads per PR. The value for read pairs with 10 nt overlap (yellow) can serve as a measurement for the intensity of ping-pong amplification and is directly comparable across different datasets.

Supplementary Figure 4

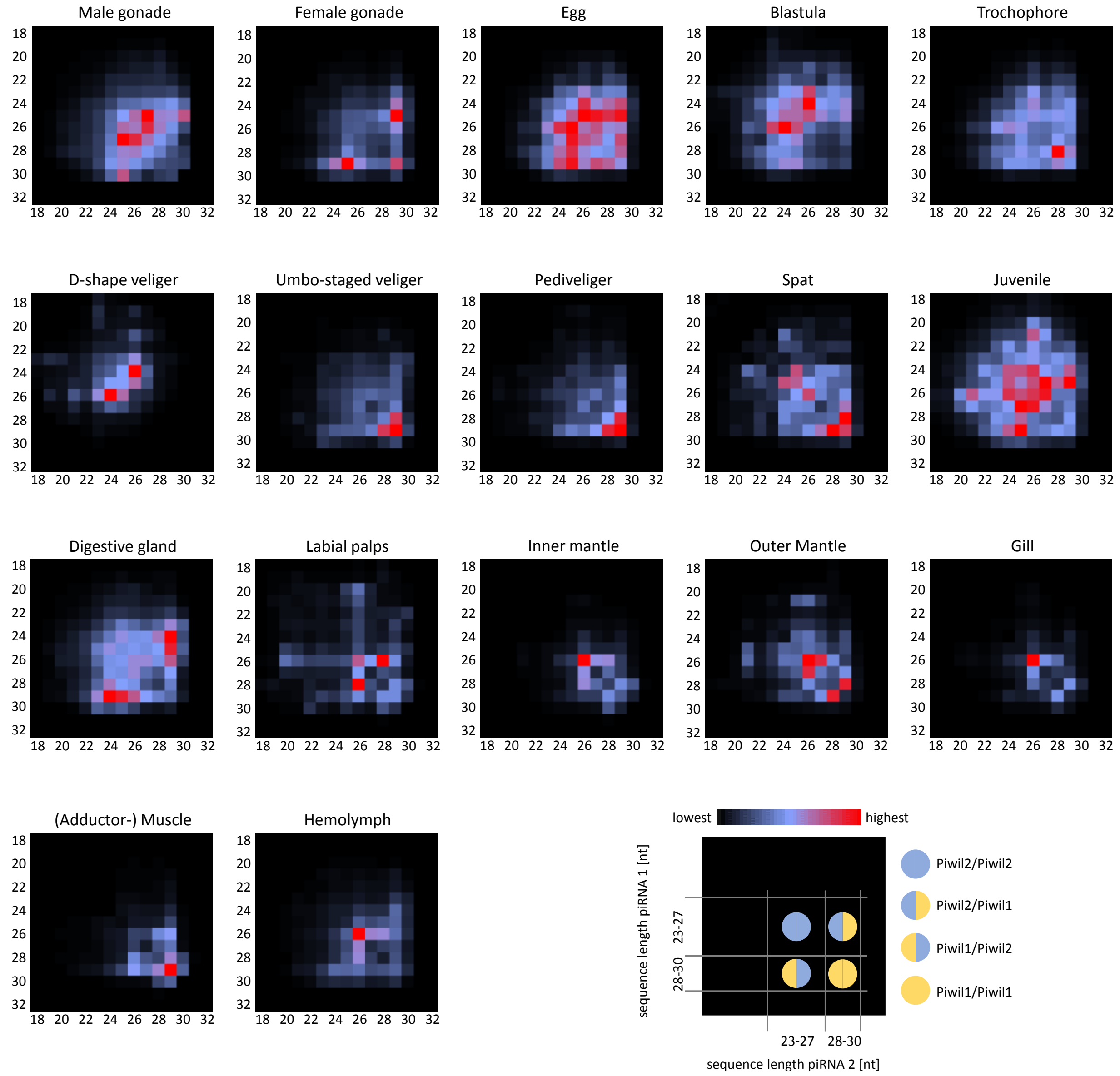

Ping-pong matrices for small RNA from different *C. gigas* samples. Frequent length-combinations of ping-pong pairs (sequences with 10 bp 5’ overlap) are indicated in red. x-axis and y-axis refer to sequence read length of the two sequences of a ping-pong pair [nt].

Supplementary Figure 5

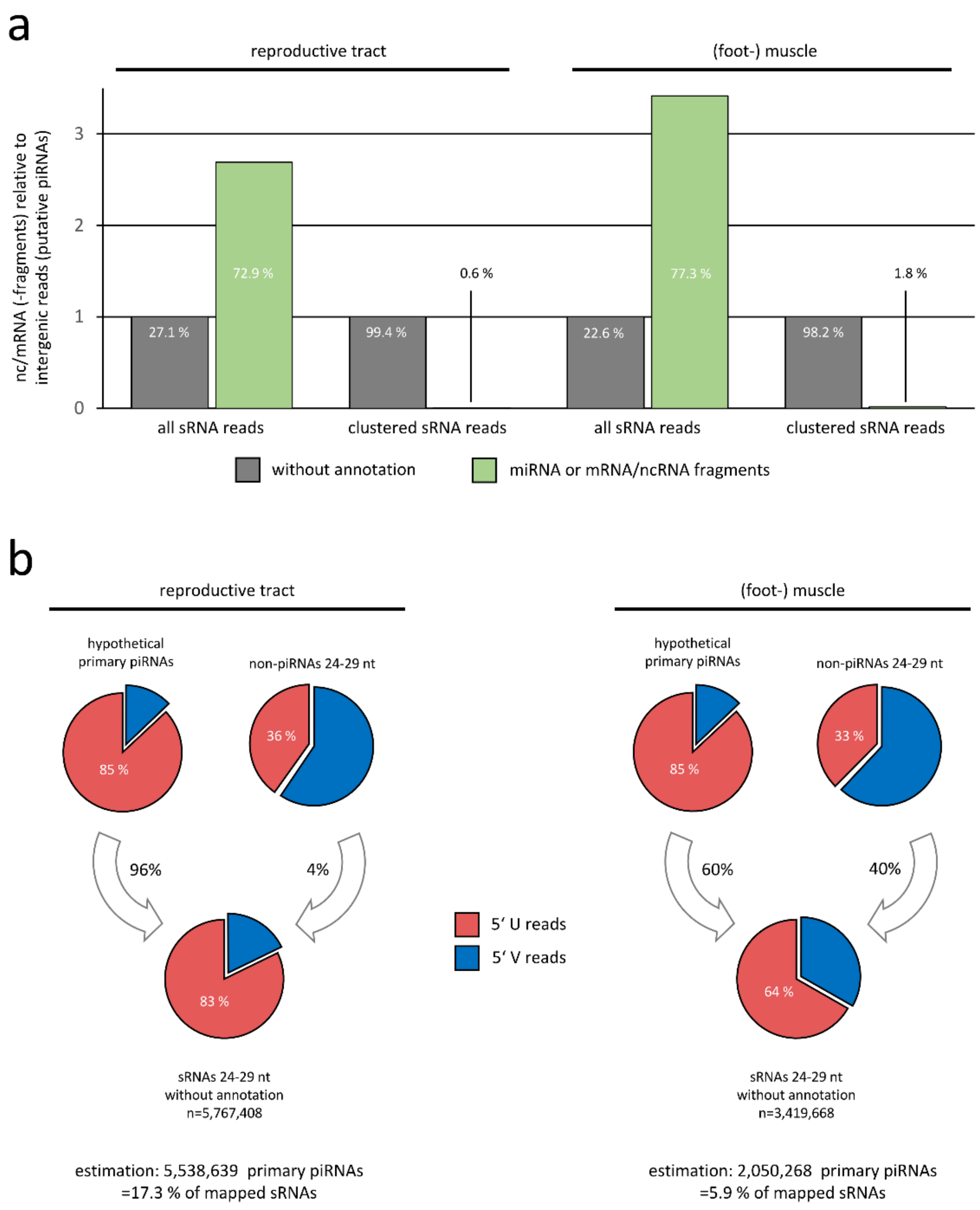

Characterization of clustered reads and alternative estimation of primary piRNA amount. (a) Fraction of reads that are either miRNAs or mRNA/ncRNA fragments regarding all sRNA reads and sRNA reads that map to piRNA clusters. (b) Estimation of the amount of primary piRNAs within the fraction of sRNA reads without annotation based on observed 1U content in 24-29 nt reads without annotation and annotated 24-29 nt reads.

Supplementary Table 1

Annotation of small RNAs from *L. stagnalis* with unitas

type	reproductive tract	muscle	reproductive tract [%]	muscle [%]
**total mapped**	32054605	34775495	100.00	100.00
**miRNA**	14668478	17804121	45.76	51.20
**rRNA**	1040112	1913871	3.24	5.50
**tRNA**	6952690	5702772	21.69	16.40
**5'tR-halves**	55077	5342	0.17	0.02
**5'tRFs**	753080	1447500	2.35	4.16
**3'tR-halves**	5081	2247	0.02	0.01
**3'tRFs**	5457457	3428816	17.03	9.86
**3'CCA-tRFs**	8954	6464	0.03	0.02
**misc-tRFs**	673041	812404	2.10	2.34
**protein_coding**	271084	537846	0.85	1.55
**snRNA**	21435	42597	0.07	0.12
**SRP_RNA**	1082	315	0.00	0.00
**snoRNA**	342	166	0.00	0.00
**ncRNA**	132	5	0.00	0.00
**RNase_MRP_RNA**	13	9	0.00	0.00
**RNase_P_RNA**	17	3	0.00	0.00
**scaRNA**	2	0	0.00	0.00
**vault_RNA**	2	0	0.00	0.00
**no annotation**	9099216	8773790	28.39	25.23
**putative parasitic contamination**	580323	1178049	1.81	3.39
**repeat sense**	1488944	1397442	4.65	4.02
**repeat antisense**	1534980	1282555	4.79	3.69
**non-repeat**	5494969	4915745	17.14	14.14

Supplementary Table 2

Annotation of small RNAs from different *C. gigas* samples with unitas.

type	male gonad	female gonad	egg	blastula	trochophore	D-shape veliger	Umbo-staged veliger	pediveliger	spat	juvenile	digestive gland	labial palps	inner mantle	outer mantle	gill	muscle	hemolymph
**miRNA**	1750589	235904	131343	490238	2447283	5454840	4074084	3451640	308870	2883310	3256489	4610791	4115836	3840707	3947487	2831842	2251774
**rRNA**	278571	136879	38225	59889	1207499	140692	65630	206196	1905155	808739	926410	299517	351470	1131787	656076	383393	133959
**tRNA**	42281	12877	6561	12743	51324	42842	71093	102552	98330	81350	86718	38098	55057	138852	80557	65008	305407
**no annotation**	4650764	5359557	5654057	5458360	5160122	1287421	1673130	1573734	662346	1169143	1688554	549725	539093	601556	708366	867161	4839829
**repeat (sense)**	991438	731730	972825	984860	1182123	327134	328575	289195	61046	332841	323065	61468	68096	84411	117016	101064	636675
**repeat (antisense)**	1411635	1815815	2121549	2129309	1805968	496982	547068	371832	60740	320661	394822	55348	71818	74194	76224	124948	1398057
**protein coding**	836511	404553	560097	537516	849040	226431	290251	247450	148820	339142	276266	66147	80364	138219	134306	98305	337002
**SRP RNA**	3627	1083	142	219	2336	742	948	1399	2615	11208	18299	5297	8505	24683	16380	6030	742
**snRNA**	1115	649	1199	1446	9124	1447	2744	3327	10059	2171	2902	953	1242	2636	2175	556	4179
**snoRNA**	274	61	72	82	1266	168	512	625	1954	572	461	220	295	553	372	127	303
**ncRNA**	84	18	8	12	156	27	121	151	256	174	151	75	76	154	88	49	154
**vault_RNA**	51	22	27	24	29	6	150	186	135	61	52	33	18	29	272	3	37
**RNase MRP RNA**	13	8	6	1	37	5	36	17	61	33	20	12	17	23	15	5	14
**nontranslating CDS**	104	113	57	59	128	25	26	23	8	44	40	8	5	11	9	5	50
**RNase P RNA**	29	5	0	0	38	7	13	7	38	15	32	11	21	26	29	15	18
**scaRNA**	3	2	0	1	9	2	7	7	10	4	4	2	1	4	3	2	1
**lncRNA**	1	0	0	0	0	0	0	0	1	0	0	0	0	0	1	0	0
**total mapped**	**9967090**	**8699276**	**9486167**	**9674760**	**12716483**	**7978771**	**7054387**	**6248342**	**3260443**	**5949467**	**6974286**	**5687704**	**5291914**	**6037843**	**5739376**	**4478512**	**9908199**

Reads without annotation or reads that match transposon (repeat) sequences represent putative piRNAs. Read counts of multiple mapping sequences were fractionated accordingly. Values are rounded, which explains possible discrepancies with the total number of mapped reads.

Supplementary Table 3

5' overlap of mRNA-matching reads from different *C. gigas* samples.

5' overlap [nt]	male gonad	female gonad	egg	blastula	trochophore	D-shape veliger	Umbo-staged veliger	pediveliger	spat	juvenile	digestive gland	labial palps	inner mantle	outer mantle	gill	muscle	hemolymph
**1**	741	201	12711	58374	19108	15174	350038	2965	0	377	279	2	1	0	1	3	139
**2**	364	525	571	1172	452	61	979	39	0	98	418	1	0	0	0	0	366
**3**	538	263	843	1348	1992	561	43179	1321	1	80	273	6	1	5	1	14	73
**4**	927	454	1584	2955	3190	3989	15734	365	1	60	257	0	0	2	1	0	101
**5**	1302	577	2047	1751	1234	104	7181	156	0	212	279	2	1	0	0	1	112
**6**	398	668	726	759	668	219	2026	184	1	84	306	1	0	0	0	3	90
**7**	1962	3310	12088	6555	10252	497	961	74	0	92	753	0	1	1	1	0	1604
**8**	2161	670	2111	2474	2301	635	4496	87	1	222	244	0	1	0	0	2	442
**9**	2128	755	2278	2421	977	597	418	10	3	76	478	0	0	1	0	0	10169
**10**	**65665**	**22281**	**44756**	**57158**	**26768**	**7908**	**4253**	**378**	**5**	**4536**	**50170**	**22**	**10**	**16**	**24**	**9**	**167909**
**11**	1355	687	1409	1263	1738	153	72	9	0	25	268	0	0	0	1	2	279
**12**	1262	634	1730	7732	1299	1418	816	154	1	257	297	0	0	1	0	4	624
**13**	900	5312	2765	2857	5098	520	18017	633	1	85	4646	2	5	11	2	1	9229
**14**	5589	3337	5842	19404	10693	13444	2184	758	1	150	59	0	0	0	0	1	58
**15**	2964	1386	2208	5560	2747	3068	2869	529	1	215	58	0	0	0	1	3	92
**16**	2471	589	723	676	466	91	278	65	8	30	66	1	0	6	1	29	174
**17**	418	345	419	432	281	85	45	81	9	33	19	0	0	1	0	0	27
**18**	979	1000	760	577	1207	143	131	36	12	41	24	1	0	0	1	1	63
**19**	962	262	250	298	152	61	84	19	7	13	27	8	3	17	115	1	162
**20**	1398	310	664	591	378	160	184	32	10	73	23	0	0	0	0	0	35

Supplementary Methods

An Arithmetical estimation of the amount of primary piRNAs

In order to support our interpretation that the number of primary piRNAs in our datasets corresponds to the number of sequence reads mapped to predicted piRNA clusters, we estimated the amount of primary piRNAs in *Lymnaea stagnalis* reproductive tract and muscle tissue with an arithmetical approach which is independent of piRNA cluster annotation. The approach is based on the assumption, that the fraction of sequence reads that do not match any other class of non-coding RNA such as rRNAs or tRNAs, contains genuine piRNAs, in addition to other RNAs that passed the annotation procedure due to either poor genome annotation, intra-species variability, post-transcriptional RNA modification or sequencing errors. To reasonably asses the amount of primary piRNAs in this mixed RNA population, we only considered molecules with a sequence length ranging from 24 nt to 29 nt as putative piRNAs, based on sequence read length profiles (Fig. 2A) and calculated ping-pong matrices (Fig. 4A and S3). Under the presumption that 85% of primary piRNAs start with a U [1,2], we argue that any deviation from 85% 5'-U reads is caused by RNA molecules other than piRNAs which can also have a U at position 1 (1U). We estimated the fraction of 1U reads in the non-piRNA population on the basis of 1U content of annotated reads in the 24-29 nt size range, ignoring tRNA fragments which were shown to preferentially bind PIWI proteins [3,4]. Using the equations below,

$$a + b = 1$$
$$a * 1U_a + b * 1U_b = 1U_{observed}$$

where *a* is the fraction of piRNAs and *b* is the fraction of non-piRNAs, $1U_{observed}$ describes the fraction of 1U reads in 24-29 nt RNAs without annotation (0.831 and 0.643 in reproductive tract an muscle, respectively), $1U_a$ describes the fraction of 1U reads in piRNAs (presumed to be 0.85) and $1U_b$ describes the fraction of 1U reads in non-piRNAs (0.359 and 0.333 for reproductive tract and muscle, respectively), we estimate the fraction of primary piRNAs to be 96,0% and 60,0% of all 24-29 nt reads without annotation in reproductive tract and muscle, respectively. These fractions account for 17.3% and 5.9% of total mapped reads in the corresponding datasets and are almost identical to the fraction of reads that map to predicted piRNA clusters, supporting the validity of piRNA cluster calling results (Supplementary Figure 5b).

Supplementary References

1. Aravin, A. A. *et al*. A novel class of small RNAs bind to MILI protein in mouse testes. *Nature*. **442**, 203-207 (2006).
2. Brennecke, J. *et al*. Discrete small RNA-generating loci as master regulators of transposon activity in Drosophila. *Cell*. **128**, 1089-1103 (2007).
3. Keam, S. P. *et al*. The human Piwi protein Hiwi2 associates with tRNA-derived piRNAs in somatic cells. *Nucleic Acids Res*. **42**, 8984-8995 (2014).
4. Honda, S. *et al*. The biogenesis pathway of tRNA-derived piRNAs in Bombyx germ cells. *Nucleic Acids Res*. **45**, 9108-9120 (2017).
