# Supplementary material for: PIWI genes and piRNAs are ubiquitously expressed in mollusks and show patterns of lineage-specific adaptation
Source: Commun Biol. 2018 Sep 7;1:137. doi: 10.1038/s42003-018-0141-4 (PMC6128900; doi:10.1038/s42003-018-0141-4)
Supplement: Supplementary file 7 — Description of additional supplementary items [file 42003_2018_141_MOESM7_ESM.docx]

**Description of additional supplementary items**

**Supplementary Data 1: piRNA clusters in *L. stagnalis.***

Chromosomal locations of each cluster and number of reads mapped to the reproductive tract and muscle tissue.

**Supplementary Data 2: piRNA clusters in *C. gigas.***

Chromosomal locations of each cluster and number of reads mapped to each tissue.

**Supplementary Data 3: PIWI peptide sequences.**

This file contains PIWI peptide sequences, a MUSCLE alignment of PIWI peptide sequences, a curated (GBlocks) MUSCLE alignment of PIWI peptide sequences and a PhyML gene tree reconstruction in NEWICK format based on the curated alignment (support values = bootstraps).

**Supplementary Data 4: Complete genome annotation in GFF3 format.**

**Supplementary Data 5: mRNA sequence file in FASTA format.**
